# Do the emotions of tourist agents contribute to improving the sustainable planning of a territory?

**DOI:** 10.3389/fpsyg.2023.1085772

**Published:** 2023-06-26

**Authors:** Rafael Robina-Ramírez, Ana Leal-Solís, Dolores Gallardo-Vázquez, Teresa Cabezas-Hernández

**Affiliations:** ^1^Departamento de Dirección de Empresa y Sociología, Universidad de Extremadura, Cáceres, Spain; ^2^Departamento de Economía Financiera y Contabilidad, Universidad de Extremadura, Badajoz, Spain

**Keywords:** emotions, tourism, participation, hotel managers, planning—market dichotomy

## Abstract

The sustainability of a territory is achieved through orderly, balanced and harmonious planning over time. Sustainable tourism planning must incorporate the emotions of interest groups. Based on a scale of negative and positive emotions that has already been validated, a participatory study of a qualitative nature has been developed with 118 hotel managers from the region of Extremadura, in the south-west of Spain. In addition, another quantitative research study has been carried out, using a longitudinal exploratory model analyzed in three phases throughout the years 2021 and 2022, using the SEM-PLS methodology. The objective is to detect if the II Tourism Plan (2021–2023) can influence the willingness of hotel managers to participate, and if this participation generates emotions that enrich the planning process of the tourist authorities. The results highlight the importance of completing decision making (cognitive part) with the measurement of emotions (sensitive part) of private agents to involve them in the planning process.

## Introduction

The specific weight of tourism as an economic reality forces administrations to design adequate land planning (UNEP, 2005; Cabezas Hernández and Leal-Solís, 2021). Planning implies coordinating chosen and designed behaviors (Leal-Solís and Robina-Ramírez, 2022), and influences how tourism develops and how its benefits and impacts are distributed (McLoughlin and Hanrahan, 2021). These behaviors can be environmental, economic, legal or social (McLoughlin and Hanrahan, 2021), contributing to the character of sustainable tourism that is observed today.

Sustainability must consider a multitude of aspects of primary importance for the territory and all the interest groups involved. However, we must be aware that the lack of environmental, social and legal planning of a destination leads to problems such as the degeneration of the natural environment, traffic problems, loss of cultural identity, lack of training of workers, conflicts between local communities and tourists, loss of tourist attraction, and many others (Dredge and Jamal, 2015).

Based on the social nature of sustainability, planning also involves learning to incorporate the people who reside in the destinations in the decision-making process (Sánchez-Oro et al., 2021), involving them through their involvement in each activity, as indicated by stakeholder theory. There are more and more studies where the population is actively involved (Baum, 2015; Robina-Ramírez et al., 2020a,b, 2021a,b; Sánchez-Oro Sánchez and Robina-Ramírez, 2020; Palazzo et al., 2021; Sánchez-Oro et al., 2021; Zheng et al., 2021). Given the importance of this group, the information that comes from the population can be communicated not only through opinions and improving the disclosure of sustainability information but also through the expression of feelings. In this sense, the generation of emotions has become an efficient tool for future decisions to be made (Hoch, 2006; Devine and Devine, 2011; Baum, 2015; Ferreira, 2018; Robina-Ramírez and Pulido-Fernández, 2021; Wang, 2021; Zheng et al., 2021; Robina-Ramírez et al., 2022).

Although planning and emotions are two phenomena that feed each other (Hoch, 2006; Deitz et al., 2018), on many occasions it is assumed that the population is not trained to make judgments that contribute to organizing the environment in which they live. For this reason, there are hardly any studies that incorporate emotions in tourism planning, and even fewer that propose an exploratory model of participatory design for the planning of a territory. Given this, our research question arises: Is there an influence of emotions in improving tourism planning?

This study aims to vindicate the role that emotions play in decision making in the tourism industry. For this, the contribution of training actions is valued as a consequence of knowledge, participation and implementation of a tourism plan. These actions are designed by public and private tourism agents to encourage the desire to participate and generate emotions.

The originality and the unique value of this work are based not only on highlighting the lack of studies that introduce emotions in the tourism planning process by public administrations but in performing training activities among the private sector in order to successfully participate in the tourism plan.

Even though the literature has proposed models for the participation of the private sector in the preparation of plans. However, this participation is usually small and unstable over time. In addition, as far as our research goes, these models have left aside the measurement of the emotions of the recipients of the tourism revitalization policies of the territories as a tool to improve planning.

Moreover, there are no empirical studies that show what type of economic, environmental, social and legal indicators should be more relevant to be trained in improving the stakeholders´ emotions towards the tourism plan, as well as training processes, that have to be performed. The originality lies not only in defining those indicators from the literature review but also in implementing innovative and sustainable actions in the five sections of the II Tourism Plan approved by the Extremadura Regional Government. In an intense effort, public administration and private companies have been aligned around the new tourism plan to propose a methodology that can be replicated in other territories.

The empirical study takes place in the region of Extremadura, located in the south-west of Spain. It is a region rich in natural parks and protected areas in 92% of the territory (Gallego and Martín, 2016). The COVID-19 pandemic reduced tourism by 2% (Engelmo-Moriche et al., 2021), which has not prevented the recovery of the sector in the post-COVID era from being outstanding, reaching 12.5% of GDP, one of the highest in Spain [INE (Instituto Nacional de Estadística), 2022].

In Extremadura, the main tourism planning instrument is three-year tourism plans. The II Tourism Plan of Extremadura (2021–2023) is currently in force, where the lines of work for that period are explained. Some authors have proposed models for the participation of the private sector in the preparation of plans (Ladkin and Bertramini, 2002). However, this participation is usually small and unstable over time. In addition, as far as our research goes, these models have left aside the measurement of the emotions of the recipients of the tourism revitalization policies of the territories as a tool to improve planning.

This study contributes to the literature from a triple approach: (1) analyzing how hotel managers can contribute to improving the tourism plan; (2) studying how the training given to hotel managers affects the strategic lines of the plan; and (3) providing a work tool for tourism authorities, by analyzing the disposition of private agents based on the emotions caused by knowledge and implementation of the plan. On the other hand, we contribute with qualitative and quantitative research to the study of sustainable tourism planning, which we consider a novelty of the study.

In the next section, the theoretical framework is mentioned, referring to the relationship between emotions and tourism planning as well as the economic, environmental, legal and social impacts caused by tourism decisions. The following sections describe the methodology and provide the results. A discussion, conclusion and limitations occupy the last three sections of the work.

## Theoretical framework

### The relationship between emotions and tourism planning: The decision-making process

Tourism planning is the pillar of any tourism organization, the sum of the interaction between its agents, the time spent, the objectives to be met and the strategies used (Robina-Ramírez et al., 2021a; Leal-Solís and Robina-Ramírez, 2022). This means that during the tourism planning process continuous collaboration between all the agents involved and the administration is required (Sánchez-Oro Sánchez et al., 2021).

In this sense, there may be divergent perspectives as to what goals should be set and how to achieve them, creating a plurality of points of view, behaviors with a different range of professionalism, mixed loyalties, moral dilemmas and other emotionally intense situations (Ferreira, 2018). An emotion is a collection of chemical and neural responses produced by the brain when detecting the presence of some stimulus, be it an object or a situation (Damasio, 2001). The role that emotions play in the tourism context has received increasing attention (Graça Ramos et al., 2021) due to the rise of the experiential paradigm in tourism (Brochado et al., 2021).

According to Wang et al. (2006), emotions are part of a person’s decision-making process (sensitive part) and knowledge (intellectual part). Emotions possess transformative (Ahmed, 2014; Moyle et al., 2019) and empathic knowledge, helping the individual to understand certain social meanings (Fischer et al., 2010). The combination of acquired knowledge and sensations is necessary to know reality (Wang, 2021), contributing value creation to personal and economic processes, in this case in the tourism sector.

Given this, the role played by emotions in the planning of tourist destinations is especially relevant due to the importance they have when it comes to attracting potential tourists (Sundaresan and John, 2020). In the tourism sector, it is essential to awaken positive emotions that channel reactions of satisfaction, affection, gratitude, happiness, well-being, enjoyment, etc. (Kim et al., 2022). These should shape the judgments made by planners when writing plans, shaping the vision one has about something (Hoch, 2006).

According to Brochado et al. (2021), economic, environmental, legal and social factors also affect the planning of a territory with a clear effect on the emotions of its recipients, and a clear contribution to sustainability. This fact can alter the process and meaning of planning decision making (Hoch, 2006; Ferreira, 2018).

### Economic impacts caused by tourism decisions

The different public agents that make decisions see tourism as an attractive sector to invest in due to the returns that these investments generate in improving the well-being and standard of living of its inhabitants (Rodríguez-Toubes and Fraiz, 2010), with an important income redistribution factor (World Travel & Tourism Council, 2013). Currently these investments are oriented towards the implementation of digitization services and infrastructures (Cabezas Hernández and Leal-Solís, 2021; Wang, 2021).

The close relationship between economic growth and tourism (Balaguer and Cantavella, 2002) causes a series of positive impacts on the taxation of a territory (Tribe, 2009). This contributes to an improvement in the balance of payments (World Travel & Tourism Council, 2013).

In contrast, decision making in economic matters can become controversial due to the massive arrival of tourists (Bec et al., 2019). This can lead to an increase in the cost of living and speculation on the value of the land (Comerio and Strozzi, 2019), which can affect the environmental impact (Bec et al., 2019). From this derive emotions that range from the state of enthusiasm and happiness to risk and fear (Mogilner et al., 2012).

### Environmental impacts caused by tourism decisions

Tourism and planning are part of a reality in which elements such as territorial, environmental and socioeconomic sustainability are integrated (Cabezas Hernández and Leal-Solís, 2021).

Changes in demand trends have been noted for years (Robina-Ramírez et al., 2020b), prioritizing the environmental paradigm over the economic one in tourist experiences (Bec et al., 2019). The areas that have exploited and mistreated their natural resources today have fewer possibilities to compete internationally (Leal-Solís and Robina-Ramírez, 2022). Thus, the competitiveness of tourist areas in the future will be based on the environmental quality they offer (Xiao et al., 2021). Tourism activity is growing with a clear conservation strategy (Mason, 2009), generating quality standards that protect the environment.

These environmental strategies also seek to generate emotional states of respect for nature (Kals and Müller, 2012; Xiao et al., 2021). Emotions can range from pleasure, excitement and satisfaction with the environment (Xiao et al., 2021) to outrage, fear and shame at the indiscriminate exploitation of nature (Kals and Müller, 2012; Nawijn and Biran, 2019; Xiao et al., 2021). Planning can harmonize not only the economic, environmental and legal interests in competition matters in a territory, but also the typology of emotions generated (Kals and Müller, 2012; Robina Ramírez and Fernández Portillo, 2020; Robina-Ramírez and Pulido-Fernández, 2021).

### Legal impacts caused by tourism decisions

Tourism, subject to a large number of plans and legal frameworks, takes into account a diversity of actors and public and private interests that are difficult to reconcile and coordinate (Cabezas Hernández and Leal-Solís, 2021). Tourism planning is the legal axis on which decisions made by public authorities revolve (Devine and Devine, 2011). Hence, collaboration between political actors and private agents is essential in the planning process (Palazzo et al., 2021). When public and private tourist agents disagree, we can say that the destination plans proceed in a contradictory way, which provokes impetuous legislative and bureaucratic modifications to satisfy all parties (Baum and Hai, 2020).

The economic strength of a country requires a system in which legal rights are guaranteed and preserved, which has a direct impact on the local tourism industry (Voigt et al., 2015). Decision making in this regard is influenced by the emotions of the moment, with indignation and shame being the most commonly perceived emotions (Vasilyev, 2017), in addition to guilt and fear (Cole et al., 2021).

### Social impacts caused by tourism decisions

The study of the emotions that derive from tourism, economic, environmental and legal planning opens the door to the study of the social impact that all planning must have (Sundaresan and John, 2020). Tourism planning must be strategic, inclusive, participatory and pluralistic to involve social, economic, environmental and legal dimensions (Leal-Solís and Robina-Ramírez, 2022).

Positive social impacts can lead to improved quality of life for residents: job creation (Mason, 2009; Tribe, 2009; World Travel & Tourism Council, 2013; Zheng et al., 2021), infrastructure improvement (Ramkissoon et al., 2013) and cultural exchange and preservation of cultural identity.

Along the same lines, the increase in leisure activities for residents also stands out (Mason, 2009; Stebbins, 2018) as well as the improvement in the conservation of cultural resources of special tourist interest (Mason, 2009). This produces a range of emotions that contribute to defining the tourist destination (Nawijn and Biran, 2019; Xiao et al., 2021), maximizing the value of the tourist experience (González et al., 2020) and improving the psychological state and satisfaction of the visitors (Stebbins, 2018; Kim et al., 2022). Consequently, the emotions aroused will be mostly positive, such as happiness, sympathy, admiration and pride (Stebbins, 2018). However, if the carrying capacity of the destination is exceeded, negative emotions such as indignation and embarrassment can occur (Xiao et al., 2021; Zheng et al., 2021).

## Methodology

### The context of the research

In January 2021, the Regional Government de Extremadura launched the II Tourism Plan (2021–2023) to determine the strategic lines of tourism for the next 3 years. The research team took advantage of this launch to analyze the response of tourism agents to the plan.

### Selection of the sample, indicators, and variables

According to the data offered by the Regional Government in Extremadura there were 146 hotels in 2021. In mid-January 2021, letters of invitation were sent to the hotel managers in Extremadura, of which 126 responded favourably. The selection of hotels was selected among the 11 tourist territories as it is considered by the Observatory of Tourism in the document “Memoria Turística de Extremadura por Territorios. Oferta y Demanda.” These touristic territories are: 1. Alqueva, Siera Suroeste, Tendudía with 13 hotels and 819 rooms. 2. La Siberia, La Serena, Campiña Sur with 14 Hotels and 469 rooms, 3. Tierra de Barros y Zafra with 33 hotels and 1839 rooms, 4. Vegas del Guadiana with 11 hotels and 563 rooms, 5. Geoparque Villuercas-Ibores-Jara, with 6 hotels and 276 rooms, 6. Reserva de la Biosfera de Monfragüe, with 11 hotels and 629 rooms, 7. Sierra de Gata, Las Hurdes, Valle del Alagón with 13 hotels and 420 rooms, 8. Tajo Internacional, Sierra de San Pedro with 14 hotels and 483 rooms, 10. Trujillo, Miajadas, Montanchez with 18 hotels and 1,085 rooms, 11. Valle del Ambroz, Tierras de Granadilla, with 11 hotels and 559 rooms. It is fair to say that the 126 favourably responses were obtained in three different ways. Among those ones, 89 responded in the first week after the letters were sent. After that 27 were reached by phone and only 11 joined the study. The rest of the hotels were visited one by one to explain in detail what the research was about and why it was important to join the research.

For the selection of the variables and indicators, the strategic lines of the II Plan and the impacts on tourism were analyzed. According to the literature, economic, environmental, social and legal indicators were studied. To select the indicators a first draft was drawn from the literature review in the areas mentioned. The research team randomly selected 11 hotels one from each tourist territory belonging to Extremadura. The commitment of each hotel was to participate in three online sessions for 1 hour to polish the first draft to elaborate the final questionnaire. After several online sessions with the selected hotel managers, 14 indicators proposed by the research team were discussed. After an online debate among the hotel managers, the five most relevant were selected. According to the hotel managers, they were the most crucial indicators to performing better results in the tourism sector (DI11–DI15) (Table 1).

**Table 1:** Hotels, departments and staff.

Hotels/Apartments	Hotel Ilunion Mérida Palace	Gran Hotel D. Manuel Cáceres	Izan Trujillo	Zurbarán Hotel Badajoz	Parador de Plasencia	Badajoz Centre Hotel
Direction	2	3	2	3	3	3
Reception	7	6	5	5	6	7
Bookings	3	3	2	3	3	2
Floors	10	12	9	9	10	9
Food and drink	6	7	6	7	6	5
Maintenance	2	2	1	2	2	1
Animation	0	1	1	2	2	1
Security	1	1	1	2	2	1
Commercial	3	3	1	2	4	3
Management	3	3	2	4	4	4
Total						

Given the relevance of territorial planning for the tourist development of the destination (Cabezas Hernández and Leal-Solís, 2021), the variable ‘DP’ (decision to participate) was defined in order to study the degree of interest of hotel managers not only in the knowledge of the II Tourist Plan but also in its response to the implementation processes by the administration and to the improvement proposal.

The second variable is ‘EG’ (emotions generated). To measure it, the negative and positive affect scale known as PANAS (Watson et al., 1988; Thompson, 2007) has been used. These emotions have been measured using the factual and counterfactual technique of Hoch (2006). Based on the proposed scales, a 10-item emotions scale was proposed to assess the typology of emotions of hotel managers when learning about, implementing and including improvements related to the II Tourist Plan. Table 2 shows the typology of emotions and their values ranging from a lower value (1 = anger) to a maximum value (10 = enthusiasm).

**Table 2:** Stress and values to reduce stress.

Values	Reduce the stress	Type of stress
Compassion		
It is a feeling generated by the suffering of others	OA3	MA
Compassion contributing to improved job performance and reduced stress	OA4	JPD
Trigger feelings of affection, care and tenderness towards others	PP1, OA1, PP2, PP3	SPA
Tolerance		
Being patience towards others opinions or practices	PP1, PP4	MA
It provides an environment of well-being in the workplace to improve the job performance.	PP2, PP3	JPD
Developing the ability to accept the values and beliefs of others	OA3	SPA
Empathy
Ability to put oneself in another’s place through the manifestation of affective and non-affective responses	PP1	MA
Better understand the situation that the person is experiencing which allow them to improve their job performance.	PP3	JPD
Empathy favours the prosocial disposition towards people reducing the stressful situations created	PP2, PP4	SPA

### Qualitative research phases

In the first week of February, a first online meeting was organized with the participants to explain the three phases of the process of including hotels in the implementation of the II Tourism Plan, as well as the strategic lines of the plan. Subsequently, the economic, environmental, social and legal implications of the plan in the territory were analysed. In the second and third phases, the answers of the hotel managers were reduced from 126 to 118. Table 3 shows the most relevant aspects of the three phases of connection with the hotel managers.

**Table 3:** Indicators.

Indicators		Authors
Values at work (VW)		
VW1	It brings strength of will to overcome any obstacle	
VW2	Improves organizational culture by providing a sense of commitment and excellence	
VW3	Help connect with workers and customers	Robina-Ramírez et al., 2021a.
Stress at work (SW)		
SW1	Improve training and confidence in dealing with the customer	Robina-Ramírez et al., 2021a.
SW2	Unforeseen changes in the hotel such as cancellations of reservations	Moreno-Luna et al., 2021.
SW3	Compliance with safety and hygiene protocols	
SW4	Uncertainty of the tourism sector	

VW1: Provides willpower to overcome any obstacle; VW2: Improves the organizational culture, providing a sense of commitment and excellence; VW3: Helps to connect with workers and clients based on values such as compassion, tolerance, and empathy; SW1: Uncertainty specific to the tourism sector; SW2: Unforeseen changes such as reservation cancellations; SW3: Compliance with safety and hygiene protocols; SW4: Improve training and confidence in dealing with the customer.

### Training sessions

In each of the phases, training sessions were given to analyse the evolution of the response of the hotel managers to the planning process. They were taught online by technicians from the General Directorate of Tourism, as well as members of the research team. There were 10 sessions of 1 hour each, with two sessions per strategic line. These themes were selected by the administration and the hotel managers in accordance with the five strategic lines of the II Tourism Plan (see Table 4).

**Table 4:** Relations between values, the development of spirituality and stressful situations.

Criteria	SDW1	SDW2	SDW3	SW1	SW2	SW3	SW4
Compassion	X	X	X	X	X	X	X
Tolerance		X	X	X	X	X	X
Emphaty		X	X		X		X
Respect	X	X	X		X		X

According to the five indicators selected by the hotel managers, 10 training actions were developed, with 1 hour each session and two sessions per indicator (see Table 5).

**Table 5:** Loadings.

	VW-01.21	VW-01.22	VW-06.21	SW-01.21	SW-01.22	SW-06.21
SW1-01.21	0.891					
SW1-01.22		0.897				
SW1-06.21			0.833			
SW2-01.21	0.823					
SW2-01.22		0.816				
SW2-06.21			0.789			
SW3-01.21	0.836					
SW3-01.22		0.898				
SW3-06.21			0.785			
SW1-01.21				0.769		
SW1-01.22					0.894	
SW1-06.21						0.894
SW2-01.21				0.802		
SW2-01.22					0.864	
SW2-06.21						0.766
SW3-01.21				0.823		
SW3-01.22					0.836	
SW3-06.21						0.884
SW4-01.21				0.796		

### Conceptual model and hypotheses

**Figure 1:**
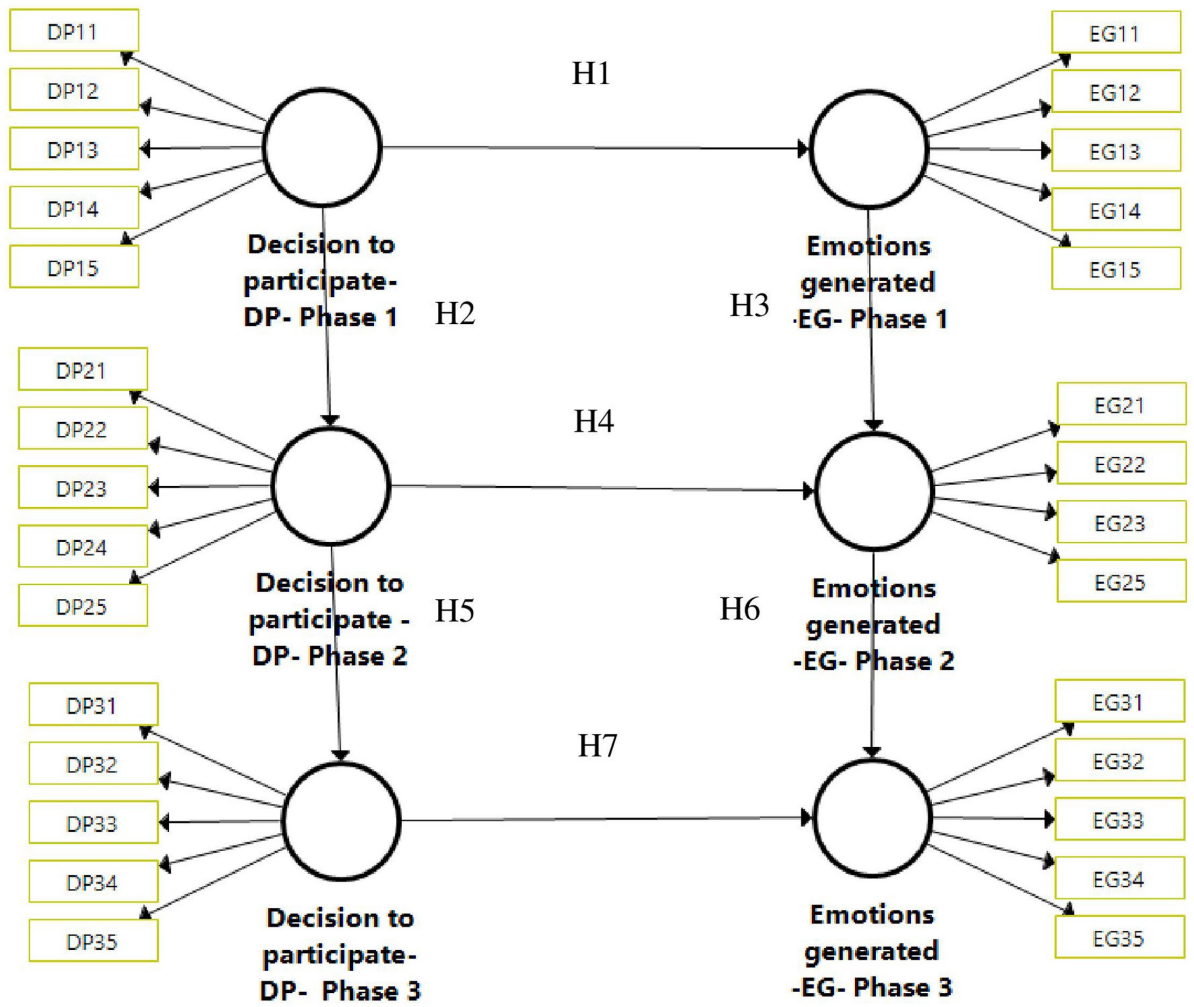
Conceptual model.

Based on the methodological design, a longitudinal study was proposed. The results of the participants are collected in three moments. According to Kim et al. (2020), the analysis of change in longitudinal data has attracted considerable attention during the last few decades in behavioral research. This is precisely because of the difficulty of extracting data from the same population at three different times. Figure 1 proposes a conceptual model developed in three different phases, where the evolution of the two latent variables (DP and EG) are studied.

In order to test the model, the following hypotheses have been defined:

*H1*. The DP in learning about the tourism plan (phase 1) generates a series of emotions (EG) for participating in the plan (phase 1).

*H2*. The DP in learning about the tourism plan (phase 1) influences the DP in defining the implications of the plan (phase 2).

*H3*. The EG for participating in the tourism plan (phase 1) generates a series of emotions (EG) in the definition of the implications of the plan (phase 2).

*H4*. The DP in learning about the tourism plan (phase 2) generates a series of emotions (EG) (phase 2).

*H5*. The DP in learning about the tourism plan (phase 1) influences the DP in defining the implications of the plan (phase 2).

*H6*. The DP in the tourism plan (phase 2) generates a series of emotions (EG) in the definition of the implications of the plan (phase 3).

*H7*. The DP in learning about the tourism plan (phase 3) generates a series of emotions (EG) (phase 3).

### Measurement model

The quantitative research deployed the multivariate partial least squares (PLS) technique to process the information obtained from the questionnaires. PLS-SEM is a well-suited exploratory methodology for small sample sizes based on normal data distribution and is well adapted for making predictions by researchers (Chin and Newsted, 1999). To generate the statistical model, SmartPLS 3 Version 26 software was applied. This version is especially recommended for explorative models (Rigdon et al., 2017).

## Results

### Qualitative research

This part of the investigation determines negative and positive emotions generated in the phases of knowledge of the plan (1st phase), implementation of proposals (2nd phase) and incorporation of improvements (3rd phase). A reduction in negative emotions (EG) and an increase in positive emotions (EGP) is observed as the plan is implemented (Kals and Müller, 2012; Vasilyev, 2017; Stebbins, 2018; Cole et al., 2021; Wang, 2021; Xiao et al., 2021; Table 6).

**Table 6:** Reliability and validity of the model.

Parameters						Forner and Larker criterion					
	AC	rho_A	CR	(AVE)		AM 20	AM 21	AM 22	WSW 20	WSW 21	WSW 22
SW-01.21	0.809	0.816	0.887	0.724	AM 20	0.851					
SW-01.22	0.842	0.857	0.904	0.759	AM 21	0.463	0.871				
SW-06.21	0.725	0.727	0.844	0.644	AM 22	0.548	0.630	0.802			
SW-01.21	0.810	0.812	0.875	0.636	WSW 20	0.593	0.507	0.588	0.798		
SW-01.22	0.832	0.841	0.899	0.748	WSW 21	0.538	0.678	0.641	0.613	0.865	
SW-06.21	0.805	0.817	0.886	0.722	WSW 22	0.679	0.682	0.620	0.589	0.682	0.850

### Quantitative research

The basic PLS algorithm follows a two-step approach: the first concerns the iterative estimation of the latent variable scores (measurement model analysis), and the second concerns the final estimation of the weights, loadings and path coefficients by means of ordinary least squares estimation (*p* > 0.05) (structural model analysis) (Henseler et al., 2015).

#### Measurement model results

In this section we analyze the reliability and validity of the model (Hair et al., 2016), first with simple correlations of the measures with their respective latent variables (λ ≥ 0.7 were accepted) (Carmines and Zeller, 1979). Table 6 shows the main parameters. The Cronbach’s alpha coefficient was used as a reliability index of the latent variables. In addition, the composite reliability was calculated. To measure validity, the mean–variance extracted (AVE) was evaluated, known as ‘convergent validity’ (accepted >0.5) (see Table 6). This was accepted because the square root of the AVE of each item exceeded the correlations with the other latent variables (Table 7).

**Table 7:** Heterotrait-monotrait ratio (HTMT).

	AM 20	AM 21	AM 22	WSW 20	WSW 21	WSW 22
VW-01.21						
VW-01.22	0.559					
VW-06.21	0.706	0.794				
SW-01.21	0.731	0.605	0.761			
SW-01.22	0.647	0.798	0.814	0.740		
SW-06.21	0.842	0.817	0.791	0.728	0.824	

Discriminant validity was verified using the Fornell-Larcker criterion (Fornell and Bookstein, 1982). Furthermore, according to Henseler et al. (2015), the heterotrait-monotrait relationship (HTMT) is the best technique to detect the lack of discriminant validity. Henseler et al. (2015) proposed testing the correlations between variables using the HTMT parameter. Since all the values are <0.90, as shown in Table 8, this condition is accepted (Henseler, 2017).

**Table 8:** Path coefficients.

Path coefficients	*β*	Confident interval (%) 2.5 97.5%		Estadísticos t (| O/STDEV|)	*p* values
H1: VW-01.21 → VW-06.21	0.548	0.468	0.641	11.997	0.000***
'H2: VW-01.21 → SW-01.21	0.593	0.501	0.681	12.657	0.000***
H3: SW-01.21 → SW-06.21	0.343	0.226	0.650	6.315	0.000***
H4: VW-06.21 → VW-01.22	0.630	0.565	0.708	17.323	0.000***
H5: SW-06.21 → SW-01.22	0.410	0.268	0.535	5.785	0.000***
H7: VW-01.22 → SW-01.22	0.399	0.267	0.556	5.273	0.000***
H6: VW-06.21 → SW-06.21	0.418	0.299	0.552	7.123	0.000***

#### Structural model results

PLS-SEM aims to maximize the amount of variance explained through the coefficient of determination (R2). The structural evaluation of the model also analyses the predictive relevance (Q2), the size and the significance of the standardized regression coefficients or path coefficients. To analyse the significance of the hypotheses, we look at the *value of p*. If the *value of p* is below its significance threshold (typically *p* < 0.05), then one can reject the null hypothesis, but this does not necessarily mean that the alternative hypothesis is true (see Table 9).

**Table 9:** Predictive and explanatory capacity.

	*R* ^2^	*Q* ^2^
VW-01.21	--	--
VW-01.22	0.397	0.296
VW-06.21	0.300	0.182
SW-01.21	0.351	0.219
SW-01.22	0.550	0.397
SW-06.21	0.462	0.324

According to Henseler et al. (2016), the best fit criterion for the global model is residual mean squared normalization (SRMR) (Hu and Bentler, 1998). A model with an adequate fit is considered when the values are less than 0.08. Therefore, a value of 0 for SRMR would indicate a perfect fit and, in general, an SRMR value of less than 0.05 indicates an acceptable fit (Byrne, 2008). A recent simulation study shows that a correctly specified model implies SRMR values greater than 0.06 (Henseler et al., 2016). Here the SRMR is 0.065, so the model is appropriate for the empirical data used (Hair et al., 2017). The R2 values (see Table 6) obtained for the investigation led to the following conclusions: 0.67 = ‘Substantial’, 0.33 = ‘Moderate’ and 0.19 = ‘Weak’ (Chin, 1998). The motivations of the older adult tourist (MT), as a dependent variable, was R2 = 65.6%. Therefore, the evidence shows that the model has moderate explanatory power. From these data, it is clear that the model has predictive capacity (Chin, 1998). Following Stone-Geisser (*Q*^2^) (Geisser, 1974; Stone, 1974), all endogenous constructs meet *Q*^2^ > 0, as can be seen in Table 9. Hair et al. (2017) also establish values of 0.02 as small, values of 0.15 as medium, and values of 0.35 as large in their predictive validity of the model. In our case, the values of DP3, DP3, EG1, EG2, and EG3 exceed the maximum threshold, indicating a high predictive relevance (Table 10).

**Table 10:** Structural model results. Path significance using percentile bootstrap 95% confidence interval (*n* = 5.000 subsamples).

H	Results	Influence	SPC	Sample mean (M)	Standard deviation (STDEV)	T Statistics |O/STDEV|	*p* Values	Change of sign
H1	Accepted ***	H1: VW-01-21 → VW-06-21	0.548	0.554	0.468	11.997	0.000	No
H2	Accepted ***	H2: VW-01-21 → SW-01-21	0.593	0.598	0.501	12.657	0.000	No
H3	Accepted ***	H3: SW-01-21 → SW-06-21	0.343	0.345	0.226	6.315	0.000	No
H4	Accepted ***	H4: SDW-06-21 → VW-01-22	0.630	0.634	0.565	17.323	0.000	No
H5	Accepted ***	H5: SW-06-21 → SW-01-22	0.410	0.405	0.268	5.785	0.000	No
H6	Accepted ***	H6: VW-01-22 → SW-01-22	0.399	0.407	0.267	5.273	0.000	No
H7	Accepted ***	H7: VW-06-21 → SW-06-21	0.418	0.419	0.299	7.123	0.000	No

## Discussion

This study is interested in the sector in which it is developed, given its contribution to the development of regions and countries. In this sense, sustainable tourism planning must be today’s objective, in order to achieve the much-desired creation of value. At the same time, the study is innovative in incorporating the emotional factor that emanates from the attitudes and actions of the agents of interest in the development of the planning of tourist activities. It is obvious that the emotions generated in said implementation have facilitated the generation of intentions to introduce improvements. In this sense, the study determines negative and positive emotions that are generated in the three phases: knowledge of the plan (1), implementation of proposals (2) and incorporation of improvements (3). The range of emotions generated at different moments of tourism planning stands out. It is observed that, as the plan is better known and shared, negative emotions (EG) are reduced (Kals and Müller, 2012; Vasilyev, 2017; Cole et al., 2021; Xiao et al., 2021) and positive ones increase EGP (Stebbins, 2018; Wang, 2021; Xiao et al., 2021). This positive turn on emotions is desired in any study on behavior, being crucial in the behavior of the final recipient (González et al., 2020; Graça Ramos et al., 2021; Kim et al., 2022) and improving the quality of the product or service provided to the customer (Table 11).

**Table 11:** Significance test results.

Effect	Path coeff (*β*)	Size of change	Confidence intervals (CI)	Comparison of Path coefficient t + 1 with CI t and path coefficient t with CI t + 1	Path coefficient t + 1 Inside CI t? Path coefficient t inside CI t + 1?	Significant change?
VW-01-21 → VW-06-21	0.548	0.173	(0.468; 0.641)	0.468 < 0.593 < 0.641	No	No
VW-06-21 → VW-01-22	0.593		(0.565; 0.708)	0.525 < 0.548 < 0.708	No	
SW-01-21 → SW-06-21	0.343	0.225	(0.226; 0.650)	0.226 < 0.630 < 0.640	No	
SW-06-21 → SW-01-22	0.630		(0.268; 0.535)	0.268 < 0.343 < 0.535	Yes	No
VW-01-21 → SW-01-21	0.410	0.289	(0.501; 0.681)	0.399 < 0.681	Yes	Yes
VW-06-21 → SW-06-21	0.399		(0.299; 0.552)	0.299 < 0.466 < 0.532	No	
VW-06-21 → SW-06-21	0.418	0.266	(0.299; 0.552)	0.299 < 0.548 < 0.552	No	No
VW-01-22 → SW-01-22	0.548		(0.267; 0.556)	0.267 < 0.418 < 0.556	No	

In this way, in similar territorial environments, emotions become an instrument not only at the service of tourist experiences, as the literature to date has shown (González et al., 2020), but also in tourism planning.

Planners are increasingly moving towards tourism as a viable economic and environmental development strategy for the territories, due to the ongoing restructuring of tourist destinations. On the one hand, in emerging tourist destinations, residents are exposed to low tourism rates, as would be the case in Spain in Extremadura, while in mature destinations the volume of tourists poses a severe risk to the lifestyle and well-being of residents. as would be the case of Spanish destinations such as the Costa del Sol, Costa Brava, Mallorca, Ibiza, or the Canary Islands. The challenge for planners in both cases is to develop a high degree of awareness of the public perception of tourism to attract the cooperation of local residents in local tourism initiatives (Lindblom et al., 2020). Planning the knowledge of emotions among hosts influences their attitudes toward dealing with guests. Previous empirical studies in the International Tagus Natural Park located within the region of Extremadura (Spain) have justified the importance of dealing with host and guest emotions in tourism planning. This host-guest relationship not only generates the tourist experience but develops reciprocal host-guest attitudes (Robina-Ramírez et al., 2020a,b). As a result, careful planning of the tourism providers´ activity help them to empathize with tourists by creating emotional bonds through developing host-guest contact points (Volo, 2017).

Those emotional bonds are analysed in the Andean community of Taquile Island (Peru) by delving into the importance of community integration planning in tourism connected to improving residents’ feelings towards tourists. By empowering locals to make decisions on socio-economic issues, residents improved their feelings towards tourists and the destination in general (Mitchell and Reid, 2001). Similarly, Hasani et al. (2016) studied the effect of incorporating emotions in tourism planning referring to the study carried out among residents in rural areas of Malaysia. The result was the incorporation of training actions among locals to improve empathy, behavior, and understanding through a project called emotional solidarity towards tourists.

Starting from the role that emotions play in the planning of tourist destinations (Sundaresan and John, 2020), the study confirms that planning and emotions are two phenomena that feed each other (Hoch, 2006; Deitz et al., 2018). The results help to overcome the uncertainties caused by currents of thought that consider that the population is not qualified to make judgments about the environment in which they live (Sánchez-Oro et al., 2021), reinforcing the influence of interest groups on the behaviour of organizations.

## Conclusions, limitations, and future research lines

The study allows us to extract numerous implications, both theoretical and practical.

The importance of a longitudinal study around sustainable tourism planning is greater now that the uncertainty of social facts is evident. According to the results, the training activities organized by the research team served to improve the willingness to participate in the II Extremadura Tourism Plan (2021–2023). The study confirms that the variation of constructs from 2021 to 2022 was significant in all cases. At the same time, it is shown that all the defined indicators were accepted as valid and reliable (*λ* ≥ 0.7). This shows that the development of infrastructures and digitization of services in the territory—improving environmental quality, defining models of public–private collaboration and reducing bureaucracy—have been key to the implementation and improvement of the plan among hotel managers throughout the three phases of the longitudinal study.

All the working hypotheses were shown to be significant. Especially strong are the relations between the variables of H1 (PD-1st Phase → EG, 1st Phase) and H4 (PD-2nd Phase → EG, 2nd Phase), H5 (PD-2nd Phase → PD-3rd Phase) and H6 (EP, 2nd Phase → EP-3rd Phase). This shows that, at the beginning of the investigation, the detailed explanation of the tourism plan generated emotions, in this case positive, among the hotel managers (H1), as happened in the process of implementing said plan (H4). The willingness to implement the project motivated the willingness to incorporate H5 improvements (DP-2nd Phase → DP-3rd Phase).

As the research progressed, the willingness of hotel managers improved. That is, the exogenous variables contributed with a greater explanatory capacity to the endogenous variable (R2-DP2 = 0.029, R2-DP3 = 0.397), as well as the perception of emotions (R2-EG1 = 0.395, R2-EG2 = 0.505, R2-EG3 = 0.517). According to Cohen (1998), the predictive capacity of the model must be >0, whether it is small (0.02), medium (0.15) or large (0.35). We observe that it is positive in all values, going from medium (Q2-DP2 = 0.020, Q2-DP3 = 0.276, Q2-EG1 = 0.264) to large, especially between the emotions generated in phases 2 and 3 (Q2-EG2 = 0.351), Q2-EG3 = 0.361).

In relation to the theoretical implications, there are three conclusions: (i) The considerable predictive capacity of the model, especially in the last year (R2 SB = 0.361), incorporates the value of emotions in the tourist planning processes of the territories. (ii) The study highlights the role of training actions, explaining the II Tourism Plan (2021–2023) to hotel managers structured in three phases: explanation of the strategic lines and selection of indicators, implementation of measures related to said indicators and incorporation of improvements after the implementation processes. (iii) Knowledge of the economic, environmental, social and legal implications of the tourism plan generated emotions in hotel managers, in line with what was expressed by Baum (2015), framed in the context of tourism sustainability of the territory. These emotions can alter the process and meaning of decision-making during planning (Hoch, 2006; Ferreira, 2018), so it would be necessary to take them into account.

We also find four empirical implications: (i) The study justifies that for the generation of positive emotions in hotel managers it is necessary to promote private sector–public sector cooperation, that is, continuous collaboration between all the agents involved and the administration (Sánchez-Oro Sánchez et al., 2021). (ii) Through the three phases—knowledge–implementation–improvements—the hotel managers contributed to complete the tourist planning from the public authority to influence the behavior of the final recipient of the service (Graça Ramos et al., 2021). (iii) In any planning process, it is necessary to complete the cognitive part with the sensitive part (Wang et al., 2006), since emotions have a transformative role when knowing reality (Ahmed, 2014). This has been revealed in the evolution of negative and positive emotions of hotel managers throughout the study. (iv) For decades, planning has taken place without coordination among the participating agents (Palazzo et al., 2021), but there are reasons to bet on alternative planning models in which emotion plays an important role (Hoch, 2006).

Although the work provided interesting results and derived numerous implications, it is not without limitations. The main limitation was the difficulty in contacting each of the hotel managers. Most have not collaborated with the regional administration to date in making tourism decisions. Sometimes this fact shows a distancing from the administration for various reasons: lack of information, lack of interest, mistrust, etc. In relation to this last fact, many hotel managers were suspicious about the benefits of their contributions to the tourism sector, so if they do not perceive results, they do not collaborate. Another limitation is that this was a regional study, carried out in a single region of Spain, derived from the publication of a regional tourism plan. There is no doubt that some results can be extrapolated to other regions, although they may differ when based on other regional tourism plans. In any case, this study is an approach to the incorporation of emotions in tourism planning.

In relation to future lines of research, and in line with the study’s limitations, it would be interesting to replicate this study in other regions, not only in Spain, but also in other countries. The objective would be to compare the relevance of knowledge of tourist plans on willingness to participate and the emotions that are generated.

## Data availability statement

The original contributions presented in the study are included in the article/supplementary material, further inquiries can be directed to the corresponding author.

## Author contributions

All authors listed have made a substantial, direct, and intellectual contribution to the work and approved it for publication.

## Funding

The publication of this work was possible thanks to the funding provided by the European Regional Development Fund and by the Consejería de Economía, Ciencia y Agenda Digital from Junta de Extremadura through grant GR21161.

## Conflict of interest

The authors declare that the research was conducted in the absence of any commercial or financial relationships that could be construed as a potential conflict of interest.

## Publisher’s note

All claims expressed in this article are solely those of the authors and do not necessarily represent those of their affiliated organizations, or those of the publisher, the editors and the reviewers. Any product that may be evaluated in this article, or claim that may be made by its manufacturer, is not guaranteed or endorsed by the publisher.
